# Resonance Raman and Visible Micro-Spectroscopy for the In-Vivo and In-Vitro Characterization of Anthocyanin-Based Pigments in Blue and Violet Flowers: A Comparison with HPLC-ESI- MS Analysis of the Extracts

**DOI:** 10.3390/molecules28041709

**Published:** 2023-02-10

**Authors:** Silvia Bruni, Margherita Longoni, Camilla Minzoni, Martina Basili, Ilaria Zocca, Stefano Pieraccini, Maurizio Sironi

**Affiliations:** Dipartimento di Chimica, Università degli Studi di Milano, 20133 Milan, Italy

**Keywords:** anthocyanins, blue flowers, natural pigments, Raman spectroscopy, visible spectroscopy, SPE, HPLC-ESI-MS, DFT calculations

## Abstract

Microanalysis techniques based on resonance Raman and reflection visible spectroscopy have been applied to the characterization of pigments responsible for the blue or violet coloration in flowers; in particular of *Lobelia erinus*, *Campanula portenschlagiana*, *Cineraria*, *Viola tricolor*, *Anemone coronaria*, *Agapanthus, Platycodon, Salvia farinacea, Plumbago capensis, Ceratostigma plumbaginoides*, *Commelina communis* and *Salvia patens*. The spectroscopic methods were applied both in vivo on the flower petals and in vitro on extracts obtained through a procedure based on SPE (solid-phase extraction) optimized for minimal quantities of vegetable raw material. Different patterns obtained for the Raman spectra have been correlated, also on the basis of density functional theory (DFT) calculations, with different schemes of substitution of the benzopyrilium nucleus of the anthocyanins and with various possible forms of copigmentation responsible for the stabilization of the blue color. The results obtained were verified by comparison with the analysis of the extracts by HPLC-ESI-MS (liquid chromatography-electrospray ionization-mass spectrometry).

## 1. Introduction

The color of the flowers is mainly due to the presence of various compounds, such as flavonoids, carotenoids and betalains [1]. In particular, among the most common flavonoids are anthocyanins, a class of compounds soluble in water and responsible for the red, purple and blue colors found in their petals [2]. Nowadays these compounds have also acquired considerable importance in the food industry, both for the natural coloring they are able to provide and for their beneficial biological properties. In fact, anthocyanins exhibit antioxidant properties, which make extracts containing them largely exploited to improve the nutritional quality of food and beverages. In addition, various protective and beneficial functions are attributed to these molecules thanks to their anti-inflammatory, anti-aging and anti-free radical capabilities [3]. Anthocyanins have also been studied to evaluate the ripening stage of grape and the organoleptic properties of wines [4]. Anthocyanins have also played an important role in the field of cultural heritage. In ancient times, in fact, vegetable extracts containing these coloring compounds were exploited to impart blue and purple dye to textiles (as still happens today in certain geographical areas) [5] and as watercolors in illuminated manuscripts [6]. In the case of traditional Japanese woodprint block, the dye obtained from *Commelina communis* was commonly used to obtain blue hues [7]. Because of their multiple applications, it is therefore interesting to be able to identify and characterize the compounds of this class.

**Figure 1:**
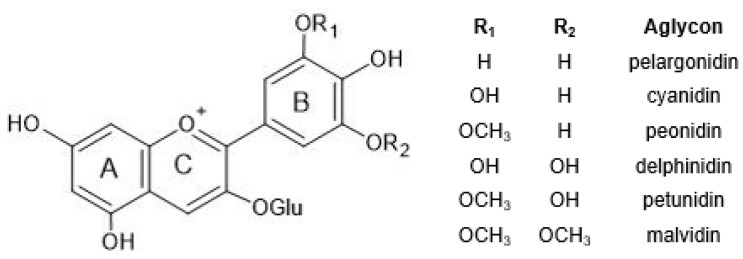
Typical structure of common anthocyanins (flavylium cation form) and their aglycons (anthocyanidins).

The chemical structure common to all anthocyanins consists of the glycosylated form of the flavylium ion (2-phenylbenzopyrilium), to which various hydroxy (−OH) and methoxy (−OCH_3_) groups are then linked (Figure 1). Their aglycones are called anthocyanidins, and twenty-three of them, differing mainly in the substitution pattern of hydroxyl and/or methoxyl groups on the phenyl ring B, have been identified in fruit and vegetables. The six most common anthocyanidins are listed in Figure 1.

**Figure 2:**
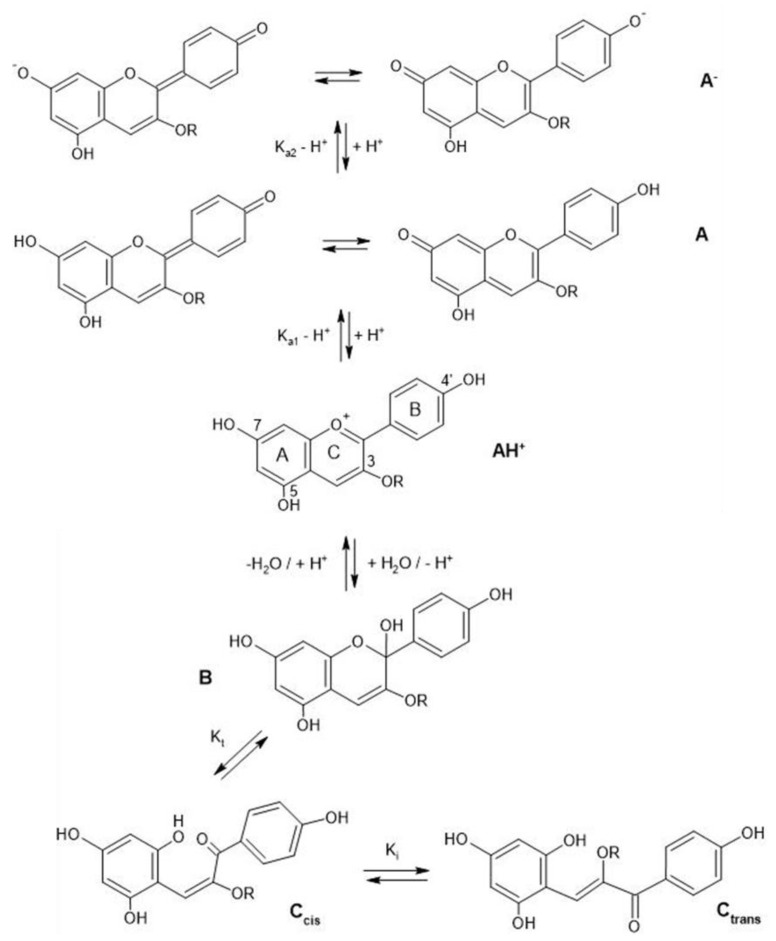
Structural variations of anthocyanins as a function of pH.

In addition to cationic form flavylium (AH^+^) in which anthocyanins are commonly represented, they can exist in various colored and colorless forms, mainly depending on pH conditions. From a general point of view, in aqueous solutions the flavylium AH^+^ form (red) predominates at very low pH values, typically lower than 2. When the pH rises, two different mechanisms are possible: deprotonation of the most acidic OH groups (those linked to C7, C5 and C4’) to form the different tautomers of the quinonoid neutral base A (purple), that prevails at pH 6-7, followed at pH > 7 by a further deprotonation forming the anionic quinonoid bases A^−^ (blue); and hydration, to form the colorless hemiketal form B, in equilibrium with the *cis*-chalcone tautomer C_cis_, which in turn is in equilibrium with the *trans* isomer C_trans_ (Figure 2) [8].

Thus, even if the word “anthocyanin” comes from the Greek “anthos” (flower) and “kyáneos” (blue), in weakly acidic or neutral media, corresponding to the pH conditions commonly observed in flower petal cells, only a purple color should be observed due to anthocyanins, and it would easily fade. A complex process should therefore be hypothesized to explain the stability of the blue color in flowers and, more generally, the great diversity of hues observed in plants due to these pigments [2,8]. Such a process is the so-called copigmentation, consisting of the formation of non-covalent complexes between an anthocyanin (most often based on delphinidin) and a copigment. The copigment should have generally an extended π-conjugated system, favoring π-π stacking interactions with the anthocyanin, and also functional groups, such as OH and C=O, suitable for hydrogen bonding. These features are mainly manifested by flavonoids (especially flavones and flavonols), which give rise to intermolecular copigmentation, and by phenolic acids, which are often linked to the pigment through the acylation of a glycosyl group, thus allowing intramolecular copigmentation. Anthocyanins can also coordinate metal ions, such as Mg^2+^, Ca^2+^ and Fe^3+^, leading to the formation of metalloanthocyanin supramolecular complexes, in which copigments also participate [2,8].

The most common methods for the analysis of anthocyanins, also applied to the study of the origin of color in blue and purple flowers, are based on the extraction of the pigments, most often with weakly acidified alcoholic solvents, and their subsequent purification, followed by the application of spectroscopic techniques. The most widely used of these techniques are UV-visible absorption spectroscopy and mass spectrometry, nowadays usually coupled with high-performance liquid chromatography (HPLC-MS) and exploiting an electrospray ion (ESI) source [9]. For the elucidation of the molecular structure, UV-visible spectroscopy is a simple technique which gives useful information concerning the substitution pattern of the benzopyrilium ion, in terms of the hydroxyl groups and their methylation or glycosilation, as well as the acylation of the sugar units [9,10,11]. Although more complex and above all expensive, ^1^H and ^13^C nuclear magnetic resonance (NMR) spectroscopy has also been extensively used, in one- and two-dimensional as well as nuclear Overhauser effect (NOE) spectroscopic experiments [9]. It should be emphasized that both UV-visible and NMR spectroscopy are most often applied to acidic solutions of the anthocyanins, where only the flavylium ion exists, in order to avoid the copresence of several colored and colorless forms which occurs at higher pH values. Furthermore, it is worth remembering that often only limited quantities of anthocyanins are available, as they are obtained from lengthy procedures of extraction from natural sources or chemical synthesis [8].

UV-visible spectroscopy has also been the technique most widely used to study copigmentation in solution, as this phenomenon gives rise to a hyperchromic effect and a batochromic shift of the electronic absorption observed in the spectra. NMR has been applied to study the stacking interaction between pigment and copigment, while circular dichroism (CD) has made it possible to distinguish between intramolecular copigmentation and chiral stacking of the chromophores [8,12]. Finally, X-ray diffraction (XRD) has been used to elucidate the structure of two metalloanthocyanins that could be obtained in crystalline form, namely commelinin from *Commelina communis* [13,14] and protocyanin from *Centaurea cyanus* [15].

It is interesting to consider those analyses that have been performed in vivo on the pigments in blue flower petals, as this approach avoids the difficulties associated with the extraction and purification procedures and the possibility that the pigment structure will be altered as a consequence of the extraction itself. The first and most easily applied technique is again UV-visible absorption spectroscopy, both in transmission and reflection mode, also associated with the determination of colorimetric parameters [16]. In one of the earliest applications, microspectrophotometry was used to correlate the color of the epidermal cells with their pH value [17]. Furthermore, in several instances the visible absorption spectra acquired in vivo have been compared with those obtained from the reconstruction in vitro of the systems which presumably give rise to the color of the petals of given flowers. It is the case of the visible spectra of acylated derivatives of delphinidin in solution at specific pH values compared with those of differently colored petals of various cultivars of *Lobelia erinus* [18], or of combinations of the anthocyanin violanin and the glycosylated flavone swertisin in varying proportions compared with the petals of different cultivars of Dutch iris [19], or again of metalloanthocyanins in several flowers [16].

Raman spectroscopic, being based on vibrational transitions, is a technique with a high degree of specificity towards molecular structures. Furthermore, it is in principle non-invasive, as scattered radiation is measured and not the transmitted or reflected one. Finally, since in most cases visible or NIR radiation is used to observe the Raman effect, optical microscopy systems allow for micro-analysis [20]. Raman spectroscopy has long been widely applied to biological molecules, as described in several books and papers (see for example [21,22,23]). To overcome the intrinsic weakness of the Raman effect, at least the competition with fluorescence emission, special techniques have been developed, in particular resonance Raman (RR) and surface-enhanced Raman (SERS) spectroscopy. The resonance conditions, verified when the selected excitation wavelength corresponds to an absorption band of the molecule of interest, allow a significant intensification of some Raman signals of the molecule itself, in particular those associated with the vibrational modes of the chromophore [21]. In addition to increasing the sensitivity of the technique, it also improves its selectivity, in principle allowing examination of a given species within complex matrices. SERS can currently be applied to solutions where the molecules are adsorbed on or close to metallic (mainly silver) nanostructured surfaces, leading to the enhancement of Raman bands thanks to an electromagnetic and, to a lesser extent, a chemical mechanism [24].

As regards anthocyanins, relatively few applications of Raman spectroscopy are reported in the literature. Among investigations on these pigments in solution, the following should be mentioned: the very first work by J. C. Merlin [25,26], in which RR spectroscopy was applied to anthocyanidins and their glycosides in acidic solution; two other RR studies of anthocyanins extracted from grape skin at different pH values [27] and the enhancement of the thermal stability of cyanidin- and delphinidin-3-glucoside by metal cations and polysaccharides [28]; an FT-Raman investigation of anthocyanin-metal interactions [29]; and SERS studies of anthocyanidins [30] and of anthocyanin extracts from berries [31] and vegetables [32]. As regards in-vivo studies, RR spectroscopy was applied by Merlin himself to the skin of *Vitis vinifera* ‘Pinot noir’ berries and the epidermal tissues of the flowers of *Malva sylvestris*, containing, respectively, malvidin 3-glucoside and 3,5-diglucoside [33], while FT-Raman was used for the in-situ characterization of pigments in flower petals of pansy cultivars of different colors [34].

The aim of the present work was to investigate the possibility of applying micro-Raman spectroscopy, with an excitation wavelength close to the resonance conditions, to the in-vivo characterization of pigments in blue and purple flowers. To verify if the spectra obtained were actually representative of the anthocyanin pigments contained in the petals, they have been compared with those acquired from the same pigments isolated from the flowers and analyzed in solution. It should be remarked that, for micro-Raman analysis, a very small amount of pigment is required, therefore an extraction and purification procedure based on solid-phase extraction (SPE) has been optimized, which allows this amount to be obtained from limited quantities of flowers. The identification of the isolated colored compounds was performed using HPLC-ESI-MS, based on the comparison with literature data. Visible reflection micro-spectrophotometry was also applied on intact petals to compare the results of in-vivo analyses carried out using the two spectroscopic techniques.

In particular, the analyzed flowers include those of: *Agapanthus*, *Anemone coronaria*, *Campanula portenschlagiana*, *Cineraria* (*Senecio cruentus*), *Lobelia erinus*, *Platycodon*, *Salvia farinacea* and *Viola tricolor*, whose color is due to *anthocyanins* based on delphinidin glycosides acylated with phenol acids [18,35,36,37,38,39,40,41,42]; *Commelina communis* and *Salvia patens*, which owe their blue color to *metalloanthocyanin supramolecular* complexes [13,43] Ceratostigma plumbaginoides and Plumbago capensis, whose flowers contain rare anthocyanins which show O-methylation on A ring, and have a blue color presumably stabilized by intermolecular copigmentation with flavonoids [44,45]. From the point of view of classification, most of the flowers examined belong to dicotyledon plants, and particularly to the clade of superasterids, except for *Anemone coronaria* and *Viola tricolor*, which belongs to the clade of superrosids. In the first group, four plants, *Campanula portenschlagiana*, *Lobelia erinus*, *Platycodon* and *Senecio cruentus*, are part of the same order of *Asterales*, and the first three of them belong to the same family of *Campanulaceae*. *Salvia farinacea* and *Salvia patens* are members of the family of *Lamiaceae*, which again is a part of the asterid clade. *Ceratostigma plumbaginoides* and *Plumbago capensis* are also included in the group of superasterids and belong to the same family of *Plumbaginaceae*. Finally, both *Agapanthus* and *Commelina communis* are instead monocotyledons, belonging respectively to the families of *Amaryllidaceae* and *Commelinaceae* [46].

For better interpretation of Raman spectra, particularly in the case of acylated delphinidin glycosides, density functional theory (DTF) calculations of the vibrational modes of 3,5-di-O-methyldelphinidin and 3,7-di-O-methyldelphinidin as model molecules have also been performed.

## 2. Results and Discussion

The flowers examined are summarized in Table 1, which also reports the results of the HPLC-ESI-MS analysis of the anthocyanin fraction extracted from the petals as described in Section 3.2. In positive ionization mode, the molecular ion [M]^+^ was usually obtained, while in negative ionization mode the deprotonated molecular ion [M-2H]^−^ was generally observed, in some case in the form of its H_2_O adduct, consistent with what is reported in literature for anthocyanins [47]. The corresponding *m*/*z* values were used to confirm the identity of the extracted pigments, by comparing them with literature data on the same flowers, also listed in Table 1. The results of the spectroscopic analyses performed both in vivo and in vitro are described and discussed below.

It should be noted that all Raman spectra were acquired with an excitation wavelength of 457 nm. This value is close to, but not exactly corresponding to, resonance conditions for the molecules examined. Indeed, as already shown in some previous studies [25,27], this choice contributes to limiting the fluorescence background which partially hides the Raman signals when using an excitation wavelength closer to the absorption maximum of the molecules and, moreover, allows more intense bands to be obtained thanks to the dependence of the Raman scattering intensity on the fourth power of the excitation frequency.

### 2.1. Blue Flower Petals Containing Acylated Delphinidin Glycosides

**Figure 3:**
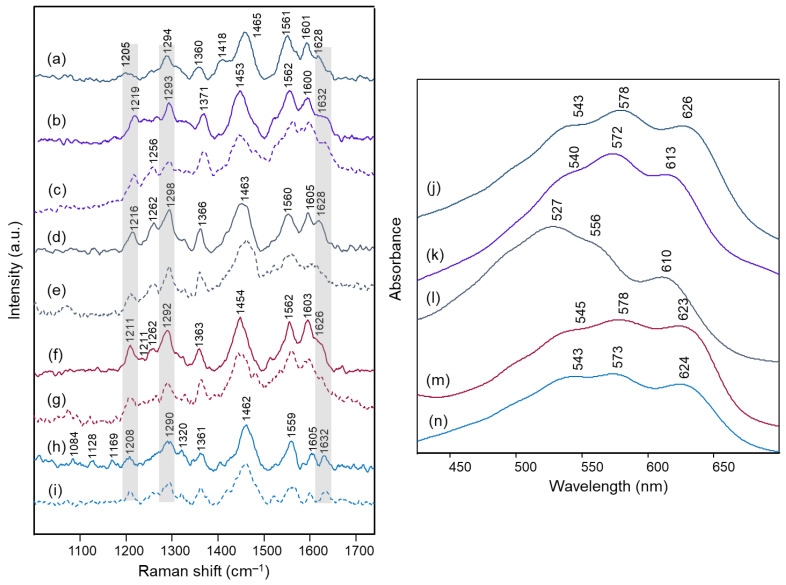
Micro-Raman spectra (λ_exc_ 457 nm) of: (**a**) *Agapanthus* intact petals; (**b**) *Anemone coronaria* intact petals and (**c**) anthocyanin extract; (**d**) *Campanula portenschlagiana* intact petals and (**e**) anthocyanin extract; (**f**) *Cineraria* intact petals and (**g**) anthocyanin extract; (**h**) *Platycodon* intact petals and (**i**) anthocyanin extract. Visible spectra acquired in microscopic reflection mode on intact petals of: (**j**) *Agapanthus*; (**k**) *Anemone coronaria*; (**l**) *Campanula portenschlagiana*; (**m**) *Cineraria*; (**n**) *Platycodon*. For easier interpretation, the same line color was used for all spectra corresponding to the same flower. Anthocyanin extracts were examined in aqueous solution at a pH value ranging from 4.5 to 6. The grey bars highlight the bands that were found to be characteristic of the 3,7-di-O substitution pattern in the benzopyrilium moiety. Legend: (solid lines) petals; (dashed lined) anthocyanin extract.

**Figure 4:**
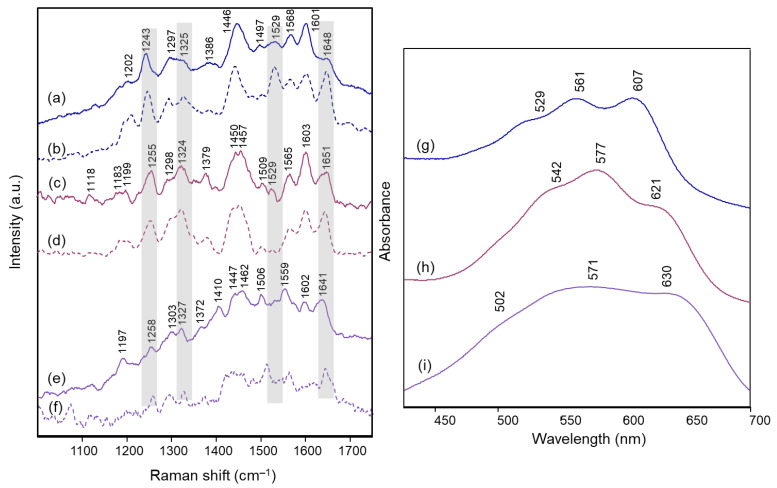
Micro-Raman spectra (λ_exc_ 457 nm) of: (**a**) *Lobelia erinus* intact petals and (**b**) anthocyanin extract; (**c**) *Salvia farinacea* intact petals and (**d**) anthocyanin extract; (**e**) *Viola tricolor* intact petals and (**f**) anthocyanin extract. Visible spectra acquired in microscopic reflection mode on intact petals of: (**g**) *Lobelia erinus*; (**h**) *Salvia farinacea*; (**i**) *Viola tricolor*. For easier interpretation, the same line color was used for all spectra corresponding to the same flower. Anthocyanin extracts were examined in aqueous solution at a pH value ranging from 4.5 to 6. The grey bars highlight the bands that were found to be characteristic of the 3,5-di-O substitution pattern in the benzopyrilium moiety. Legend: (solid lines) petals; (dashed lined) anthocyanin extract.

Figure 3 and Figure 4 show the Raman spectra obtained on the intact petals and on the isolated anthocyanin fraction of each flower. In the same Figure 3 and Figure 4, the visible absorption spectra recorded on the petals are also reported.

The visible absorption spectra show in all cases the three components already observed in solution at pH values of 4 and almost 7 for polyacylated delphinidin pigments [11], at about 530–540, 560–570 and 610–620 nm. This pattern has been assigned to the coexistence of the flavylium and quinonoidal forms in solution, with the neutral A form prevailing at the lower pH value and the A^−^ form gaining importance at the higher one. In the present work, for all the flowers examined the component around 560–570 nm is the most intense, apart from the flower of *Campanula*, for which the component at the lower wavelength predominates. All spectra resembled those obtained in transmission for the anthocyanin extracts in aqueous solution at pH 4.5–6 (Figure S1, Supplementary Material). In the case of *Campanula*, a trend more similar to that of the flower could be observed only at a pH value lower than 4 (Figure S2, Supplementary Material). It can be noted that, different from what is reported in the literature for visible spectra acquired at very acidic pH, in the pH conditions typical of petals (usually ranging from 3 to 8 [8,11]) the visible absorption spectrum appears to be less easily associated with differences in the substitution scheme of the anthocyanin.

On the other hand, considering the resonance Raman spectra, it should first of all be highlighted that an excellent correspondence was found in all cases between those acquired on the petals and on the aqueous solutions at pH 4.5–6 of the extracted anthocyanin pigments (the latter spectrum is not shown for *Agapanthus* due to its poor signal-to-noise ratio). This means that the experimental conditions are suitable to isolate the response of these pigments from that of other species, colored or colorless, also contained in the petals. Furthermore, unlike what is observed above for visible absorption spectra, the resonance Raman spectra showed two distinct patterns, one shown in Figure 3 and relating to those flowers for which the color is due to acylated 3,7-di-O-glycosyldelphinidins (also in mixture as for *Anemone coronaria* and *Platycodon*) and the other one, shown in Figure 4, to those for which the pigment (or sometimes mixture of pigments, as in the cases of *Lobelia erinus* and *Salvia farinacea*) was an acylated 3,5-di-O-glycosyldelphinidin (in the case of *Salvia farinacea* a derivative of malvidin is also present). In fact, besides common bands around 1600, 1560, 1450 and 1370 cm^−1^, the hydrogen substitution of the OH groups in positions 3 and 7 gives rise to more evident bands signals at about 1630, 1290 and 1210–1220 cm^−1^, while the substitution in positions 3 and 5 favors bands at 1650–1640, 1530 (less evident in the case of *Viola tricolor*), ca. 1325 and 1255–1245 cm^−1^. The more or less extended chains of glycosyl and acyl units linked to the anthocyanin core seem to have only minor effects on the observed spectra. 

To verify the proposed interpretation and to assign the Raman bands, DFT calculations of the vibrational frequencies were performed for two model molecules, i.e., the 3,5-di-O-methyldelphinidin and the 3,7-di-O-methyldelphinidin, where the two methyl groups replace the glycosyl units of the real molecules. For both molecules, three forms were considered, i.e., the flavylium cation AH^+^ and the two tautomers of the neutral A form (Figure 2), respectively the 4’- and 7-quinonoidal ones for the first molecule and the 4’- and 5-quinonoidal ones for the second molecule [11]. In Table 2 and Table 3 the calculated and experimental Raman wavenumbers are reported. 

**Figure 5:**
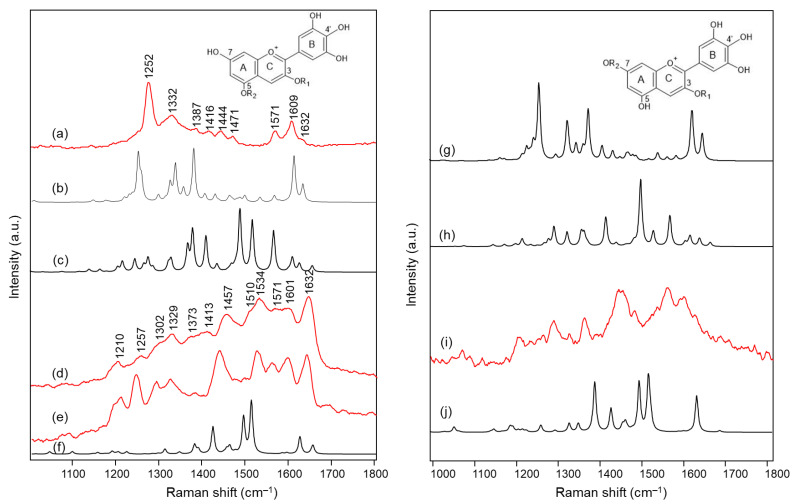
(**a**) Experimental FT-Raman spectrum of solid delphinidin 3,5-diglucoside chloride; (**b**) calculated Raman spectrum of 3,5-di-O-methyldelphinidin, AH^+^ form; (**c**) calculated Raman spectrum of 3,5-di-O-methyldelphinidin, 4’-quinonoidal A form; (**d**) experimental Raman spectrum (λ_exc_ 457 nm) of delphinidin 3,5-diglucoside in aqueous solution at pH 4; (**e**) experimental Raman spectrum (λ_exc_ 457 nm) of the anthocyanin extract from flowers of *Lobelia erinus* (for the peak wavenumbers see Figure 4); (**f**) calculated Raman spectrum of 3,5-di-O-methyldelphinidin, 7-quinonoidal A form; (**g**) calculated Raman spectrum of 3,7-di-O-methyldelphinidin, AH^+^ form; (**h**) calculated Raman spectrum of 3,7-di-O-methyldelphinidin, 4’-quinonoidal A form; (**i**) experimental Raman spectrum (λ_exc_ 457 nm) of the anthocyanin extract from flowers of *Cineraria* (for the peak wavenumbers see Figure 3); (**j**) calculated Raman spectrum of 3,7-di-O-methyldelphinidin, 5-quinonoidal A form. Legend: (black lines) calculated spectra; (red lines) experimental spectra.

In detail, for the 3,5-di-O substituted delphinidin, the values calculated for the AH^+^ flavylium cation are compared with the normal FT-Raman spectrum of the solid delphinidin 3,5-glucoside chloride. A very good correspondence is obtained, as also shown in Figure 5a,b. Instead, the experimental values measured for the flowers and their anthocyanins are compared with the vibrational wavenumbers calculated for the 4’-quinonoidal form of A, considering the pH conditions of the petal cells and the aqueous solution of the extracts and also the visible spectra discussed above. As apparent in Figure 5, the contribution of the 7-quinonoidal form of A to the experimental spectra seems less important. It should be noted, however, that the experimental spectra of the flower petals and the related anthocyanins were obtained in conditions close to resonance ones, favoring bands related to the chromophore, while the calculated values obviously refer to the normal Raman spectra. Consistent with this, based on the DFT calculations, the experimental bands are in most cases assigned to vibrations of the benzopyrilium moiety of the molecules, associated with vibrational modes of the B ring (Table 2). For example, the strong calculated band at 1380 cm^−1^ has no correspondence in the experimental spectra and indeed is mainly attributed to the bending modes of the C‒OH bonds (Table 2). For the same reason, as previously observed, the Raman spectra acquired on these flowers and the related polyacylated anthocyanins are not significantly affected by the peripheral substituents.

In the case of the 3,7-di-O-substituted delphinidin, as expected a markedly different pattern is calculated compared to the substitution in positions 3 and 5 especially for the 4’-quinonoidal A form (Figure 5h), even more evident than for the AH form (Figure 5g). Also in this case the bands observed in the experimental resonance Raman spectra (in Figure 5i the spectrum at pH 6 of the anthocyanin cinerarin extracted from flowers of *Cineraria* is shown for comparison) correspond to vibrations of the benzopyrilium nucleus and the phenyl B ring, while for example the strongest band calculated, located at 1495 cm^−1^ and due to a mode with a significant contribution of δ(OH) vibrations (Table 3), appears poorly enhanced in the RR spectra. 

### 2.2. Blue Flower Petals Containing Metalloanthocyanins

**Figure 6:**
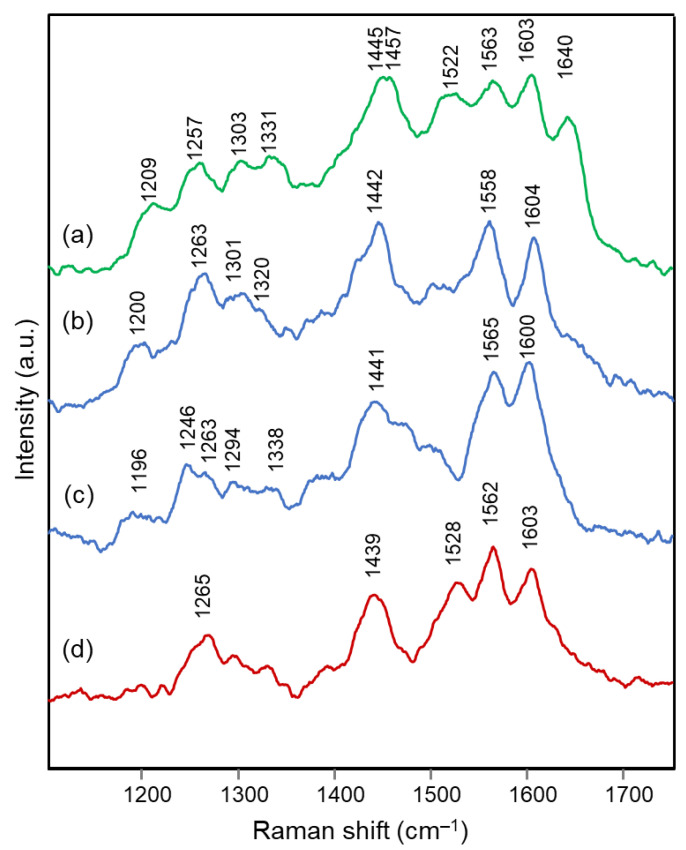
Micro-Raman spectra (λ_exc_ 457 nm) of: (**a**) malonyawobanin in aqueous solution at pH 4; (**b**) intact petal of Commelina communis; (**c**) reconstructed commelinin; (**d**) intact petal of Salvia patens.

The resonance Raman spectra of the flowers of Commelina communis and Salvia patens are shown in Figure 6.

As reported above, according to the literature [13,43,50], the blue color of both flowers is due to a supramolecular complex containing six molecules of the anthocyanin maloylawobanin and six molecules of a flavone coordinated to two Mg^2+^ ions. In the case of Commelina the flavone is the so-called flavocommelin (7-O-methylapigenin 6-C-4’-O-di-glucoside), while for Salvia patens it is apigenin 7,4’-di-O-glucoside. 

It is important to highlight that malonylawobanin is in fact an acylated 3,5-di-O-glucosyl delphinidin (Table 2) and, according to T. Kondo [13], in the two complexes, named respectively commelinin and protodelphin, the 4’-quinonoidal form of the deprotonated anthocyanin is stabilized. 

**Figure 7:**
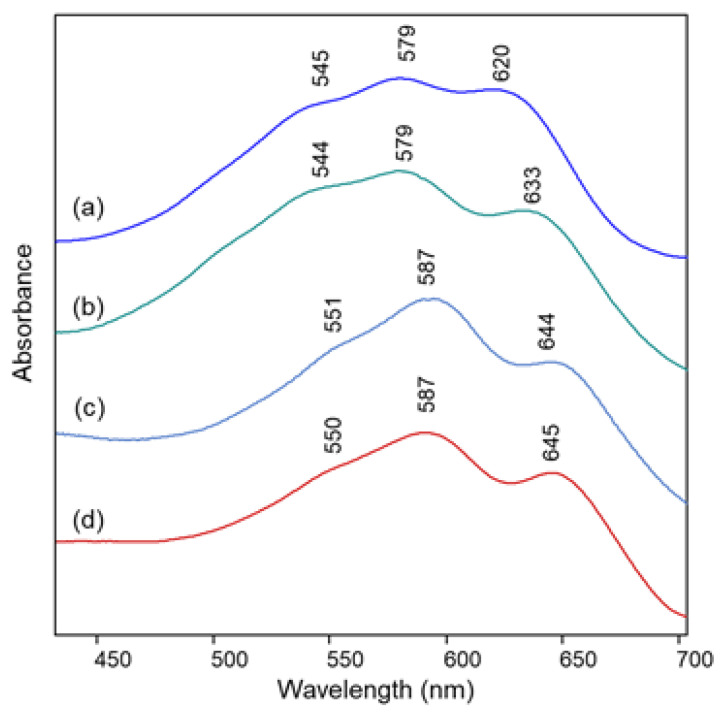
Visible spectra acquired in microscopic reflection mode on intact petals of: (**a**) Ceratostigma plumbaginoides; (**b**) Plumbago capensis; (**c**) Commelina communis; (**d**) Salvia patens.

In the present work the resonance Raman spectrum of malonylawobanin, extracted as described in Section 3.3, was acquired in aqueous solution at pH 4 and the observed pattern was consistent with all other similarly substituted anthocyanins examined here (Figure 6a, Table 2) and different from those of the flowers (Figure 6b,d), in which the band at 1640 cm^−1^ disappears, that at about 1450 cm^−1^ moves to 1440 cm^−1^ and the band at 1331 cm^−1^ decreases in intensity. The spectra obtained for the petals of the two flowers of Commelina and Salvia patens are instead very similar to each other, and to those of their aqueous extracts containing the undissociated complex (Figure S3, Supplementary Material), confirming that the structures of the species responsible for their color are also similar. The same result has been obtained for the visible absorption spectra of the petals (Figure 7c,d), which agree with those reported in the literature for commelinin [13] and protodelphin [43], with a maximum at 587 nm and two shoulders, the strongest of which is found at 645 nm and the other at 550 nm.

Furthermore, the reconstruction of commelinin from its components was attempted as reported again in Section 3.3 and the corresponding resonance Raman spectrum was recorded (Figure 6c). For the reconstructed complex the same variations already described are observed with respect to the spectrum of the isolated malonylawobanin. They can be interpreted by considering the band assignments reported in Table 2 for the anthocyanin. In particular, for malonylawobanin, the 1640-cm^−1^ signal is attributed to the stretching modes of the C=C bonds of the benzopyrilium nucleus and of the C=O bond of the B ring in quinonoidal form, while the band at about 1450 cm^−1^ is assigned to a vibrational mode that includes the bending of C‒O‒H bonds on the B ring. In both cases, therefore, functional groups are involved that participate in the coordination of the metal ions in the supramolecular complex and it is reasonable to observe a lowering of the corresponding vibrational frequencies. The disappearance of the signal at 1640 cm^−1^ can probably be explained by assuming that, if the corresponding wavenumber decreases, it is hidden by the band at about 1600 cm^−1^. Finally, the signal at 1331 cm^−1^, which appears weakened when passing from the isolated anthocyanin to the flowers and the complex, is assigned to a vibrational mode which includes the bending of C‒O‒H bonds of the B ring. 

In summary, it can be hypothesized that the spectral pattern observed for the flowers of Commelina communis and Salvia patens is indeed characteristic of a 3,5-di-O substituted delphinidin when complexed to a metal ion.

### 2.3. Blue Flower Petals Containing Rare Anthocyanins

The petals of the blue flowers of Ceratostigma plumbaginoides and Plumbago capensis were also examined using resonance Raman and visible reflection spectroscopy. As anticipated in the Introduction, the color of the two flowers is attributed to peculiar anthocyanins bearing methoxy groups on the A ring [44,45] and, at least in the case of Plumbago, it has been supposed that a mechanism of copigmentation with flavonols is involved in stabilizing the blue color itself [45]. In fact, the anthocyanins identified in the flowers of Plumbago lack OH groups in orto position on the B ring, which could in principle favor complexation with metal ions, and are not acylated, thus leading to the exclusion of intramolecular copigmentation.

**Figure 8:**
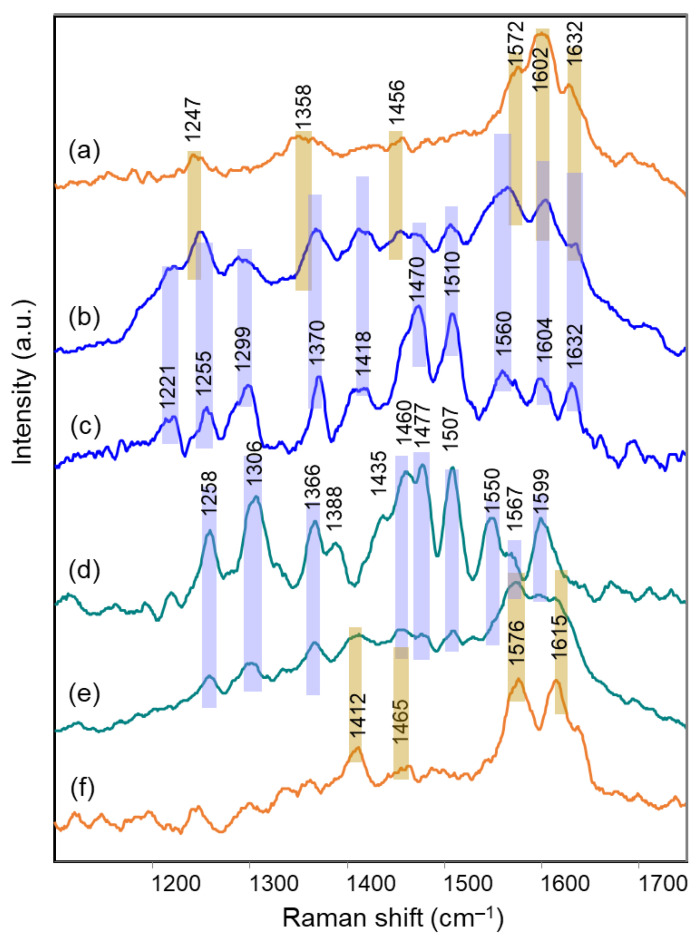
Micro-Raman spectra (λ_exc_ 457 nm) of: (**a**) flavonol fraction isolated from the petals of Ceratostigma plumbaginoides; (**b**) intact petal of Ceratostigma plumbaginoides; (**c**) anthocyanin fraction isolated from the petals of Ceratostigma plumbaginoides in aqueous solution at pH 6; (**d**) anthocyanin fraction isolated from the petals of Plumbago capensis in aqueous solution at pH 5.5; (**e**) intact petal of Plumbago capensis; (**f**) flavonol fraction isolated from the petals of Plumbago capensis. Legend: (orange lines) flavonols; (light blue lines) anthocyanins and intact petals. The vertical bars allow to recognize in the spectra of the petals the bands due respectively to flavonols and anthocyanins.

The visible absorption spectra acquired on petals of the two flowers (Figure 7a,b) show a trend very similar to those of all the blue flowers examined above. Instead, a peculiar situation is encountered when resonance Raman spectra (Figure 8b,e) are considered. Also in the case of Ceratostigma and Plumbago, in fact, the anthocyanins have been extracted from the petals and identified using HPLC-ESI-MS as reported in Table 1. Their RR bands observed in aqueous solution (Figure 8c,d) are also actually recognized in the spectra of the intact petals, for which, however, other signals are also present, sometimes more intense. This is the case of the bands at 1572 and 1456 cm^−1^ and of the shoulder at 1358 cm^−1^ for the Ceratostigma petal and of the bands at 1615, 1576, 1465 and 1412 cm^−1^ for the Plumbago flower. To interpret these differences, the Raman spectra of the flavonol fraction obtained by the purification of the pigments on DSC-MCAX cartridges (Section 3.2) were also acquired. In the case of Plumbago, the HPLC-ESI-MS analysis proved the prevalence of the flavonol azalein ([M]^+^ at *m*/*z* 462), as already suggested by J. B. Harborne [44], while for Ceratostigma the most abundant species has shown a molecular ion at *m*/*z* 495, with a fragment at *m*/*z* 333 (unidentified). The corresponding Raman spectra (Figure 8a,e) contained the bands observed for the intact petals in addition to those of the anthocyanins. This fact can obviously indicate a higher concentration of flavonols in these flowers than those previously discussed, or even a weaker resonance effect for the involved anthocyanins, but it can be considered at least indicative of the possibility that the copigmentation mechanism relies in these two cases on the intermolecular interaction of anthocyanins with flavonols. Indeed, flavonols or flavones were obviously present in all the flowers considered above, and the excitation wavelength used could give a pre-resonance effect also for these molecules, whose absorption maxima are in the near-UV range, but only for these two flowers are their Raman bands observed with significant intensity in the spectra obtained for the intact petals.

Finally, the Raman spectral pattern of the anthocyanins extracted from Ceratostigma and Plumbago (Figure 8c,d) is characteristic, different from those observed previously and similar for the two flowers. It is characterized in particular by the two strong bands at 1510 and 1470 cm^−1^ and by medium-strong bands at about 1370, 1300 and 1255 cm^−1^. The observed similarity should be discussed while considering the proposed structures for the two anthocyanins involved. In fact, the anthocyanin contained in Ceratostigma flowers should have the structure reported in Table 1 and related to europinidin (the anthocyanin suggested by J. B. Harborne for this flower [44] on the basis of hydrolysis experiments and the UV-visible spectrum). However, the anthocyanin extracted in the present work has a molecular ion [M]^+^ at *m*/*z* 493, corresponding to europinidin (as reported in Table 1) but also to 5,7-dimethyldelphinidin 3-O-galactoside according to the study on Plumbago capensis reported in [45]. On the other hand, the most abundant anthocyanin for the flower of this Plumbago species appears to be, both in the present work and in the above reference, 5,7-dimethylmalvidin 3-O-rhamnoside (Table 1) and not capensinidin (5-O-methylmalvidin 3-O-rhamnoside) as hypothesized in [44]. The similarity between the two RR spectra could indeed suggest that the blue pigments of both flowers show the same substitution pattern on the A ring, with two methoxy groups in position 5 and 7. The difference possibly arising when the involved anthocyanidin is a malvidin and not a delphinidin deserves further investigation. 

## 3. Materials and Methods

### 3.1. Materials

The flowers examined were purchased from local shops or grown in pots. Delphinidin 3,5-diglucoside was purchased from Extrasynthese (Genay, France).

Amberlite XAD-7, Sephadex LH-20 and DSC-MCAX cartridges (all Sigma-Aldrich brand, St. Louis, MO, USA) used for the purification of the extracted pigments were purchased from Merck Life Science S.r.l (Milano, Italy).

### 3.2. Extraction and Purification of Extracts

For the extraction of the pigments from the flower, a methodology has been developed that does not alter the coloring molecules. The flowers were first frozen in liquid nitrogen to minimize anthocyanin degradation and aid in the destruction of cell compartments resulting in color release, then they were ground in a mortar. The ground flowers, whose quantity varies from 0.3 to 6 g, were then dispersed in a suitable volume (from 20 to 90 mL) of the so-called MAW solution, composed of methanol (MeOH), acetic acid (AcOH) and water in a ratio of 10: 0.5: 9. This solvent has a high extractive capacity and at the same time avoids, in particular, the decomposition of acylated anthocyanins due to acid hydrolysis. The whole was sonicated for 30 min, then centrifuged for 15 min at 6000 rpm to separate the extracted flowers from the supernatant, which was collected and concentrated under gentle nitrogen flow. Subsequently, the concentrated sample was washed 2–3 times with diethyl ether to remove any organic compound soluble therein. Several extractions with MAW were repeated on the same sample in order to obtain greater quantities of anthocyanins to be used for subsequent analyses. The described procedure was adopted for all the flowers with the exception of *Commelina communis* and *Salvia patens*, for which the extraction was carried out in Milli-Q water to avoid the destruction of the supramolecular complex from the petals.

For the purification of the extracts, a solid-phase extraction (SPE) process consisting of three different step was performed, as described below:

1. SPE on a cartridge packed with Amberlite XAD-7: the sample was eluted from the cartridge using 3 mL of (A) Milli-Q H_2_O: AcOH 20: 1 and 3 mL of (B) MeOH: AcOH 20: 1. The A fraction contains free sugars and organic acid molecules, while pigment molecules were eluted with the B fraction. The cartridge was pre-conditioned in the same way. This step was performed for all the flowers except for *Commelina communis* and *Salvia patens*.
2. SPE on a cartridge packed with Sephadex LH-20: the eluents were (A) MeOH: H_2_O:AcOH in a ratio 6:12:1 and (B) MeOH. The extracts loaded into the cartridge were fractionated using eluent A and 2 to 4 fractions were collected depending on the sample. By eluting with B, a final yellowish fraction containing flavonols and/or flavones was obtained. Conditioning was performed using 3 mL of B. This step was carried out as described for all the flowers except for *Commelina communis* and *Salvia patens*, for which the supramolecular complex was eluted with Milli-Q H_2_O so as not to cause alterations.
3. SPE with DSC-MCAX cartridge: (A) Milli-Q H_2_O + 0.1% HCOOH, (B) MeOH and (C) 0.01 M phosphate buffer solution at pH 6 and MeOH in the ratio 1:1 were used as eluents. The sample was dissolved in a small amount of A, loaded and washed with A. The residual flavonols/flavones were then eluted with B and finally the anthocyanins with C. Conditioning of the cartridge was achieved with 3 mL of B and 2 mL of A.

### 3.3. Reconstruction of Commelinin

The supramolecular complex of commelinin was prepared for comparison from its components, following the procedure described in the literature [51].

Due to the limited quantity of flowers available for commelin, an extraction from a sheet of Japanese paper colored with this flower was carried out to obtain the starting anthocyanin and flavone. The process consisted of a preliminary step of extraction in Milli-Q water followed by SPE on Amberlite XAD-7. The solvents used were first H_2_O: MeOH and then Milli-Q water to elute the complex. This fraction was dried, subsequently dissolved in a few mL of a solution composed of H_2_O + 10% TFA and sonicated for about half an hour to dissociate the complex: after this step the solution turns red. The components were separated using a DSC-MCAX cartridge: H_2_O + 20% MeOH + 1% TFA was used to elute the flavone flavocommelin and H_2_O + 60% MeOH + 0.5% TFA for the anthocyanin malonylawobanin [52]. Their identity was checked through HPLC-ESI-MS analysis.

After drying under nitrogen flux, the anthocyanin was dissolved in a little water and treated with 0.5 M ammonia until a color change from pink to purple/blue was observed, ensuring that the pH was between 4 and 6. This step serves to obtain the carboxylate anion of the anhydrous base. The anthocyanin was then dried and weighed and the required quantities of potassium acetate (CH_3_COOK) and magnesium acetate tetrahydrate ((CH_3_COO)_2_Mg ∙ 4H_2_O) were prepared to have an anthocyanin:flavonol:K:Mg ratio of 1:1:1:2. The flavocommelin was dissolved in 200 μL of Milli-Q water and the anthocyanin fraction was added. Subsequently the two salts were added and the solution thus obtained was stirred at room temperature for about half an hour until a color change from violet to blue was observed, indicating the formation of the complex commelinin. To isolate the complex, ethanol was added as reported in the literature, so that after a few days a blue precipitate was obtained.

### 3.4. Instrumental Analysis Techniques

#### 3.4.1. Raman Spectroscopy

Micro-Raman resonance (RR) spectra were acquired using a Jasco TRS 300 triple monochromator spectrometer (2400 lines/mm grid) (Jasco Europe, Cremella, Italy) equipped with an Andor CCD detector (Andor Technology Ltd., Belfast, UK) and interfaced with an Olympus BH-2 microscope (Olympus, Tokyo, Japan), provided with three different objectives (10×, 20× and 50×). A Cobolt Twist TM 25 laser (Cobolt, Stockholm, Sweden) with emission at 457 nm and maximum power of 25 mW was used as excitation source. For each analysis the spectral region 1000–1800 cm^−1^ was considered and the spectra were acquired as the sum of 60 accumulations with an exposure time of 2 seconds.

The Raman spectra were corrected using the Savitsky-Golay smoothing algorithm and the baseline correction by means of the Grams/AI software (Thermo Fisher Scientific, Waltham, MA, USA).

The Fourier-transform (FT) Raman spectrum of delphinidin 3,5-diglucoside chloride was acquired using a Jasco RFT-600 spectrophotometer (Jasco Europe, Cremella, Italy), equipped with a Nd-YAG laser (1064 nm). The output laser power was 100 mW. The spectrum was recorded as sum of 300 accumulations with resolution 4 cm^−1^.

#### 3.4.2. HPLC-ESI-MS

HPLC-MS analyses were performed using a Thermo Fisher LCQ Fleet ion trap mass spectrometer equipped with the UltiMate™ 3000 UPLC system with ESI source and UV detector (Thermo Fisher Scientific, Waltham, MA, USA). The column used was an Agilent ZORBAX RX-C18 column (5 µm, 2.1 × 150 mm) (Agilent Technologies Italia, Milano, Italy), thermostated at 35 °C and with an operating pressure of 53 MPa. The wavelengths set for the UV-Vis detection system were 380 and 500 nm. The eluents were (A) H_2_O milliQ + 0.01% HCOOH and (B) acetonitrile + 0.1% HCOOH and the following gradient was used: 0’, 90% A–10% B; 2’, 90% A–10% B; 50’, 100% B; 55’ 100% B; 60’, 90% A–10% B. 

#### 3.4.3. Visible Absorption Micro-Spectroscopy

Visible reflection (on petals) and transmission (on extracted pigments in solution) analyses were performed using a portable micro-probe equipped with an Olympus 20 × objective and connected via optical fibers to a halogen source (maximum power 150 W) and to a Lot Oriel MS125 spectrometer (400 lines/mm grid) (Quantum Design, Rome , Italy) provided with an Andor CCD detector (1024 × 128 pixel) cooled by means of a Peltier device (Andor Technology Ltd., Belfast, UK). The micro-probe was equipped with a beam splitter 30/70 for the 400–700 nm spectral range. The wavelength calibration was based on the emission spectrum of a neon lamp. The spectra were acquired as the sum of 30 scans with an exposure time of 0.01 s at the maximum laser power. In the case of petals, the analysis was performed in reflection mode directly on them, while for extracts a micro-drop of sample (10 µL) was deposited on a coverslip glass slide and placed on a mirror. The analyses were preceded by the acquisition of a background spectrum in the absence of the incident radiation and of a reference, which was a metal target coated with barium sulphate or the mirror itself.

### 3.5. Quantum Mechanical Calculations

Quantum mechanical calculations were performed using the DFT approach in order to consider electron correlation effects, whose role can be particularly significant for these systems. The hybrid functional B3LYP, largely used for this kind of studies, was used. The B3LYP functional includes exchange terms based on the HF and DFT theory and a contribution of correlation based on the Lee functional [53,54]. The B3LYP functional was parametrized by Becke [55,56]. The basis set adopted was the double-zeta split valence 6-31G** [57]. Molecular geometries were fully optimized without imposing any constraints. The harmonical vibrational frequencies were evaluated at the optimized geometries and were scaled using the factor 0.9732, a typical value in the range already used in similar studies [58]. Calculations were carried out using the Gaussian 16 program (Gaussian, Inc., Wallingford, CT, USA, 2016) and the plot of the simulated spectra was obtained using GaussView 6.0.16 (Shawnee Mission, KS, USA, 2016).

## 4. Conclusions

The application of Raman spectroscopy with an excitation wavelength close to resonance conditions to the in-vivo study of the blue color in flower petals has been investigated.

The relationship between the spectra obtained in vivo and those acquired in solution at mildly acidic pH values on the pigments isolated from the flowers (whose identity was checked using HPLC-ESI-MS analysis) was first of all examined. In case of delphinidin-based polyacylated and glycosylated anthocyanins, an excellent correspondence was found in all cases, and the same result was obtained in the case of metalloanthocyanins when the Raman spectra recorded in vivo were compared with that of a complex such as commelinin reconstructed in vitro. In the case of the flowers of *Ceratostigma* and *Plumbago*, where an intermolecular copigmentation between anthocyanins and flavonols can be hypothesized, the Raman spectra acquired on intact petals showed evidence of both classes of pigments.

Considering now the dependence of the observed Raman spectra on the structure of the pigments, for polyacylated delphinidin glycosides two distinct patterns have been detected relating to the substitution of the benzopyrilium nucleus in positions 3 and 5 or 3 and 7. The two patterns have been interpreted through DFT calculations of the vibrational modes for both the flavylium cation and the quinonoidal neutral base of 3,5-di-O- and 3,7-di-O-methyldelphidin as model molecules. It is worth noting that the visible spectra obtained in reflection on the intact petals were much less sensitive to this structural difference. On the other hand, the RR spectra are obviously much more affected by the chromophore, i.e., the phenylbenzopyrilium, than by the peripheral substituents, such as the long chains of glycosyl and acyl units, that cannot be distinguished. 

Still regarding the information about the structure of the pigments provided by the RR spectra, as expected a modification was observed when metalloanthocyanins were responsible for the color of the petals, as in the case of *Commelina communis* and *Salvia patens*. Therefore, in principle, Raman spectroscopy represents a useful means to recognize in vivo the presence of these complexes.

A distinctive trend was also detected for the “special” anthocyanins contained in the flowers of *Ceratostigma plumaginoides* and *Plumbago capensis*, whose structure is characterized by the presence of methoxy groups on the A ring.

In conclusion, resonance Raman spectroscopy can be considered a useful method to characterize rapidly and in vivo the anthocyanin pigments responsible for blue (or violet) colors. This possibility is of interest as the extraction procedures and *in-vitro* analyses could in principle alter the molecules or their environment from those present in plants. The method could also be applied in other fields, e.g., the food one, where these colors are of interest.

Further investigation will be required to extend this application and, in particular, clarify the possible variations of the RR spectra associated with pigments based on anthocyanidins other than delphinidin.

## Figures and Tables

**Table 1:** Flowers whose petals have been examined in the present work using micro-Raman spectroscopy. The *m*/*z* values of the ions observed in the HPLC-ESI-MS analysis of the anthocyanin fraction isolated from the petals, both in positive and negative ionization mode, are also reported, together with the literature references based on which the pigments have been identified and the corresponding molecular structures.

Flower	HPLC-ESI-MS (*m*/*z*)	Anthocyanin	Ref.	Molecular Structure
*Agapanthus* 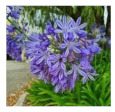	[M]^+^ 1597 [M-H]^−^ 1596	(6” ‘-O-(Delphinidin 3-O-(6”-O-p-coumaroylglucoside) 7-O-glucosyl)) (6” “-O-(kaempferol 3-O-glucoside, 7-O-xyloside, 4’-O-glucosyl)) succinate	[35]	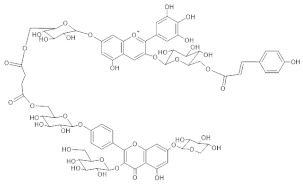
*Anemone coronaria* 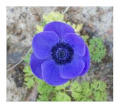	(1) [M]^+^ 1289 [M-2H]^−^ 1287 (2) [M]+ 1375 [M-2H]^−^ 1373 (3) [M]^+^ 1507 [M-2H]^−^ 1505 (4) [M]^+^ 1491 [M-2H]^−^ 1489 (5) [M]^+^ 1331 [M-2H]^−^ 1329	(1) Delphinidin 3-[2-(2-(caffeoyl)glucosyl)galactoside]-7-[6-(caffeoyl)glucoside]-3’-[glucuronide] (2) Delphinidin 3-[2-(2-(caffeoyl) glucosyl)-6-(malonyl)galactoside]-7-[6-(caffeoyl)glucoside]-3’-[glucuronide] (3) Delphinidin 3-[2-(2-(caffeoyl)glucosyl)-6-(2-(tartaryl)malonyl)galactoside]-7-[6-(caffeoyl)glucoside]-3’-[glucuronide] (4) Delphinidin 3-[2-(2-(caffeoyl)glucosyl)-6-(2-(tartaryl)malonyl)galactoside]-7-[6-(caffeoyl)glucoside] (5) Cyanidin 3-[2-(2-(caffeoyl) glucosyl)-6-(2-(tartaryl)malonyl) galactoside]-7-[6-(caffeoyl) glucoside]-3’-[glucuronide]	[36]	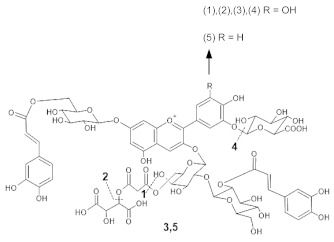
*Campanula portenschlagia-na* 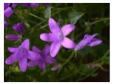	[M]^+^ 1175 [M-2H]^−^ 1173	Violdelphin [7-O-(6-O-(4-O-(6-O-(4-hydroxybenzoyl)glucosyl)-oxybenzoyl)glucosyl)-3-O-(6-O rhamnosyl-glucosyl)delphinidin	[37,48]	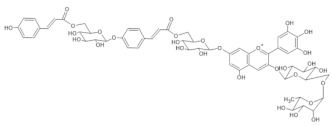
*Ceratostigma plumbaginoides* 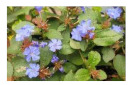	[M]^+^ 493 [M-2H+H_2_O]^−^ 509	Europinidin-3-galactoside	[44]	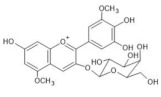
*Cineraria (Senecio cruentus)* 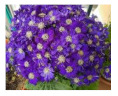	[M+H]^+^ 1523 [M-H]^−^ 1521	Cinerarin [3-O- (6-O-malonylglucosyl)-7-O-(6-O-(4-O-(6-O-caffeoylglucosyl)caffeoyl)glucosyl-3’-O-(6-O-caffeoylglucosyl)delphinidin]	[38]	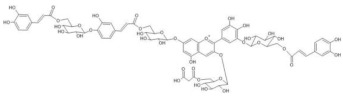
*Commelina communis* 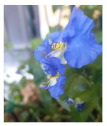	[M]^+^ 859 [M-2H+H_2_O]^−^ 875	Malonylawobanin [3-O- (6-O- (trans-*p*-coumaroyl)glucosyl) -5-O- (6-O-malonylglucosyl) delphinidin]	[49]	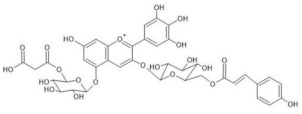
*Lobelia erinus* 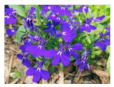	(1) [M]^+^ 1567 [M-2H]^−^ 1565 (2) [M]^+^ 1653 [M-2H]^−^ 1651 (3) [M]^+^ 1667 [M-2H]^−^ 1665	(1) Demalonyllobelinin A [3-O-(6-O-(4-O-(*p*-coumaroyl)rhamnosyl) glucosyl)-5-O-glucosyl- 3’,5’ -di-O-(6-O-(caffeoyl)glucosyl) delphinidin] (2) Lobelinin A [3-O-(6-O-(4-O-(*p*-coumaroyl)rhamnosyl) glucosyl)-5-O-(6-O-(malonyl)glucosyl- 3’,5’ -di-O-(6-O-(caffeoyl)glucosyl) delphinidin] (3) Lobelinin B [3-O-(6-O-(4-O-(*p*-coumaroyl)rhamnosyl) glucosyl)-5-O-(6-O)-malonyl)glucosyl- 3’-O-(6-O-(caffeoyl)glucosyl)- 5’-O-(6-O-(feruloyl)glucosyl) delphinidin]	[18]	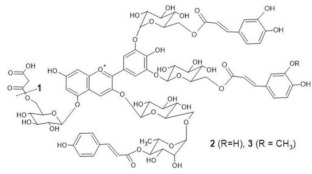
*Platycodon* 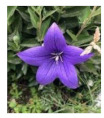	(1) [M]^+^ 1421 [M-2H]^−^ 1419 (2) [M]^+^ 1405 [M-2H]^2-^ 1403	(1) Platyconin [3-O-(6-O- (rhamnosyl)-glucosyl) -7-O-(6-O-(4-O-(6-O- (4-O-(glucosyl)- caffeoyl)glucosyl)caffeoyl)glucosyl) delphinidin] (2) 3-O-(6-O-rhamnosyl-glucosyl)-7-O-(6-O-(4-O- (6-O-(4-O-glucosyl-*p*-coumaroyl)glucosyl caffeoyl)glucosyl) delphinidin	[39,40]	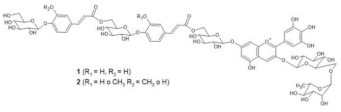
*Plumbago capensis* 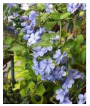	[M]^+^ 505 [M-2H+H_2_O]^−^ 521	3-O-rhamnosyl-5,7-di-O-methylmalvidin	[45]	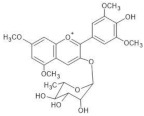
*Salvia farinacea* 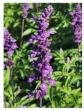	(1) [M]^+^ 859 [M-2H+H_2_O]^−^ 875 (2) [M]^+^ 887 [M-2H+H_2_O]^−^ 903	(1) Malonylawobanin (see *Commelina communis*) (2) Salviamalvin [3-O-(6-O-*p*-coumaroylglucosyl)-5-O-(6-O-malonylglucosyl) malvidin]	[41]	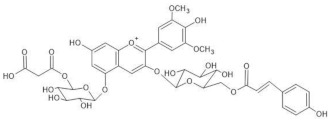
*Salvia patens* 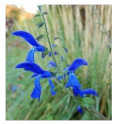	[M]^+^ 859 [M-2H+H_2_O]^−^ 875	Malonylawobanin (see *Commelina communis*)	[43]	
*Viola tricolor* 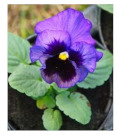	(1) [M]^+^ 919 [M-2H+H_2_O]^−^ 935	(1,2) Violanin [3-O-(6-O-(*p*-coumaroyl rhamnosyl)glucosyl)-5-O-glucosyldelphinidin]	[42]	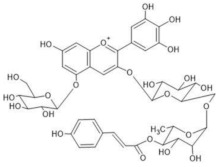

**Table 2:** Experimental and calculated Raman wavenumbers (cm^−1^) for 3,5-di-O-substituted delphinidin. The wavenumbers reported for the flowers refer both to the spectra obtained for intact petals and for the extracted anthocyanins in aqueous solution at pH values from 4.5 to 6.

Delphinidin 3,5-di-O- methyl AH^+^ Form	Delphinidin 3,5- diglucoside Chloride	Delphinidin 3,5-di-O- methyl 4’-quinonoidal A Form	Delphinidin 3,5-di- glucoside	*Lobelia*	*Viola tricolor*	*Salvia farinacea*	Malonyl-Awobanin	Assignment
Calc.	Exp.	Calc.	Exp.	Exp.	Exp.	Exp.	Exp.	
	FT-Raman (solid)		RR (aqueous solution pH 4)	RR	RR	RR	RR (aqueous solution pH 4)	
1638	1632	-	-	-	-	-	-	ν(C=C) benzopyrilium
-	-	1630 (+1613)	1650	1648	1641	1651	1640	ν(C=C) benzopyrilium + ν(C=O)
1618	1609	-	-	-	-	-		ν(C=C) B ring
1571	1571	1569	1601	1601	1602	1603	1603	ν(C=C) B ring + δ(C‒H) benzopyrilium
-	-	1520	1571	1568	1559	1565	1563	ν(C=C) benzopyrilium, B ring and inter-ring
-	-	1491	1534	1529	-	1529	1522	ν(C=C) benzopyrilium, B ring and inter-ring, δ(C‒OH) B ring
-	-	1470	1510	1497	1506	1509	1505	δ(C‒H) benzopyrilium and B ring
1465	1471	-	-	-	-	-	-	δ(C‒H) aliphatic
		1437	1457	1446	1447	1450	1445	δ(C‒H) A ring
1432	1444	-	-	-	-	-	-	δ(C‒H) benzopyrilium
-	-	1412	-	-	1462	1457	1457	ν(C=C) benzopyrilium + δ(C‒H) and δ(O‒H) B ring
1408	1416	-	-	-	-	-	-	δ(C‒OH) B ring
1381	1387	-	-	-			-	δ(C=C‒C) + δ(C‒OH)
		1380	-	-	-	-	-	δ(C‒OH) A and B rings
1357	-	-	-	-	-	-	-	δ(C‒H) C ring + δ(C‒OH) B ring
-	-	1368	1413	-	1410	-	-	breathing A ring + δ inter-ring with B ring
1338	1332	-	-	-	-	-	-	δ(C=C‒C) + δ(C‒O‒H) B ring
-	-	1330	1373	1386	1372	1379	-	δ(C=C‒C) + δ(C‒O‒H) A ring
-	-	1276	1329	1325	1327	1324	1331	δ(C‒H) + δ(C‒OH) B ring
-	-	1266	1302	1297	1303	1298	1303	δ(C‒H) + δ(C‒OH) A ring and B ring
1251	1275	-	-				-	δ(C‒O‒H) + ν(C‒O) benzopyrilium
-	-	1245	1257	1243	1258	1255	1257	δ(C‒H) benzopyrilium
-	-	1216	-	-	-	-	-	δ(C‒H) + δ(O‒H) benzopyrilium and B ring
-	-	1205	1210	1202	1197	1199	1209	δ(C‒H) + δ(O‒H) A ring

**Table 3:** Experimental and calculated Raman wavenumbers (cm^−1^) for 3,7-di-O-substituted delphinidin. The wavenumbers reported for the flowers refer both to the spectra obtained for intact petals and for the extracted anthocyanins in aqueous solution at pH values from 4.5 to 6.

Delphinidin 3,7-di-O- methyl 4’-quinonoidal A Form	*Agapanthus*	*Anemone*	*Campanula*	*Cineraria*	*Platycodon*	Assignment
Calc.	Exp.	Exp.	Exp.	Exp.	Exp.	
	RR	RR	RR	RR	RR	
1612	1628	1632	1628	1626	1632	ν(C=C) + ν(C=O) benzopyrilium
1564	1601	1600	1605	1603	1605	ν(C=C) benzopyrilium + ν(C=O) + ν(C=C) + ν B ring
1525	1561	1562	1560	1562	1559	ν(C=C) B ring + ν(C=C) benzopyrilium
1495	-	-	-	-	-	δ(C‒OH) and δ(C‒H) B ring + ν(C=C) benzopyrilium and inter-ring
1413	1465	1453	1463	1454	1462	breathing benzopyrilium + δ(C‒OH) B ring
-	1418	-	-	-	-	
1362	1360	1371	1366	1363	1361	ν(C=C) benzopyrilium and B ring + δ(C‒H)
1355	-	-	-	-	-	ν(C‒OH) A ring
1322	-	-	-	-	-	breathing A ring + δ inter-ring with B ring
1291	1294	1293	1298	1292	1290	ν(C=C) + δ(C‒H) and δ(O‒H) A ring
1215	1205	1219	1216	1211	1208	δ(C‒H) rings

## Data Availability

Data sharing is not applicable to this article, as all data are reported within it.

## Supplementary Materials

The following supporting information can be downloaded at: https://www.mdpi.com/article/10.3390/molecules28041709/s1, Figure S1: Visible spectra in aqueous solution at pH values from 4.5 to 6 of the anthocyanin fraction extracted from the flowers; Figure S2: Visible spectra in aqueous solution at different pH values of the anthocyanin fraction extracted from the flowers of *Campanula portenschlagiana*; Figure S3: Micro-Raman spectra of the aqueous extracts obtained from the flowers of *Commelina communis* and *Salvia patens*.
