# Supplementary material for: Resonance Raman and Visible Micro-Spectroscopy for the In-Vivo and In-Vitro Characterization of Anthocyanin-Based Pigments in Blue and Violet Flowers: A Comparison with HPLC-ESI- MS Analysis of the Extracts
Source: Molecules. 2023 Feb 10;28(4):1709. doi: 10.3390/molecules28041709 (PMC9959989; doi:10.3390/molecules28041709)
Supplement: Supplementary file 1 [file molecules-28-01709-s001.zip › molecules-2197316-supplementary.pdf]

Resonance Raman and visible micro-spectroscopy for the in-vivo and in-vitro characterization of anthocyanin-based pigments in blue and violet flowers: a comparison with HPLC-ESI- MS analysis of the extracts

Silvia Bruni *, Margherita Longoni, Camilla Minzoni, Martina Basili, Ilaria Zocca , Stefano Pieraccini and Maurizio Sironi

Supplementary material

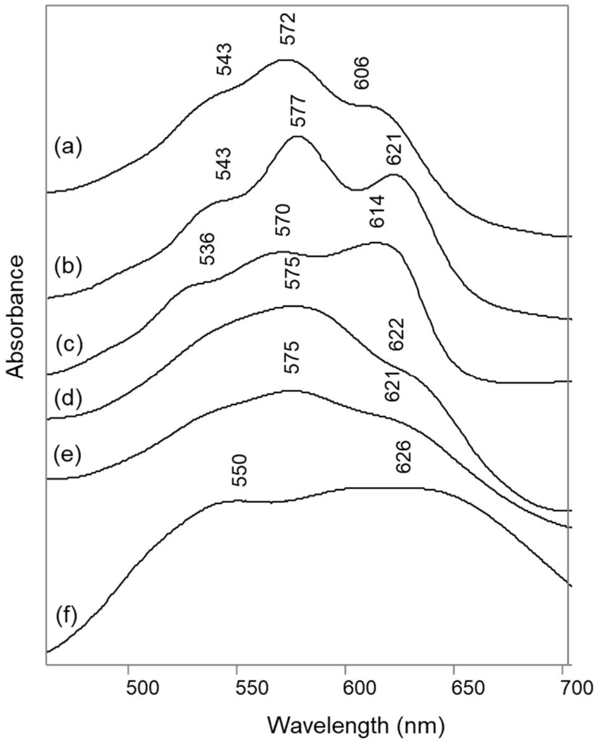

Figure S1. Visible spectra in aqueous solution at pH values from 4.5 to 6 of the anthocyanin fraction extracted from the flowers of: (a) *Anemone coronaria*; (b) *Cineraria*; (c) *Lobelia*; (d) *Platycodon*; (e) *Salvia farinacea*; (f) *Viola tricolor*.

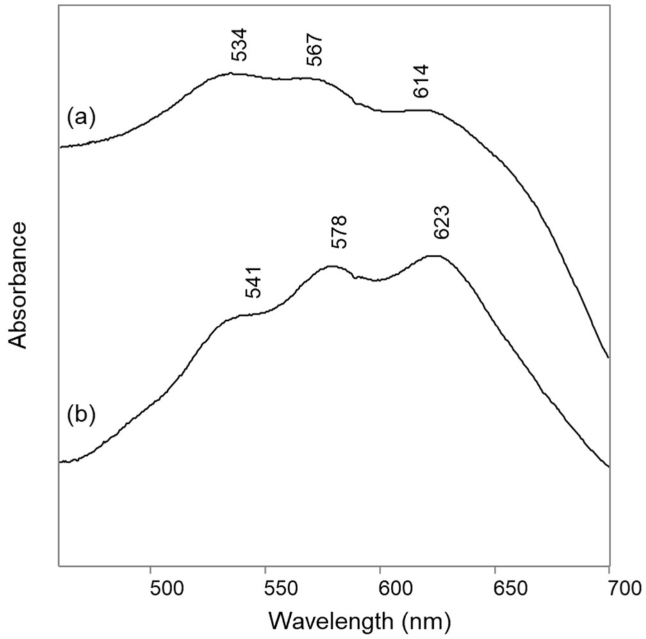

Figure S2. Visible spectra in aqueous solution of the anthocyanin fraction extracted from the flowers of *Campanula portenschlagiana*: (a) at pH 3.8; (b) at pH 6.0.

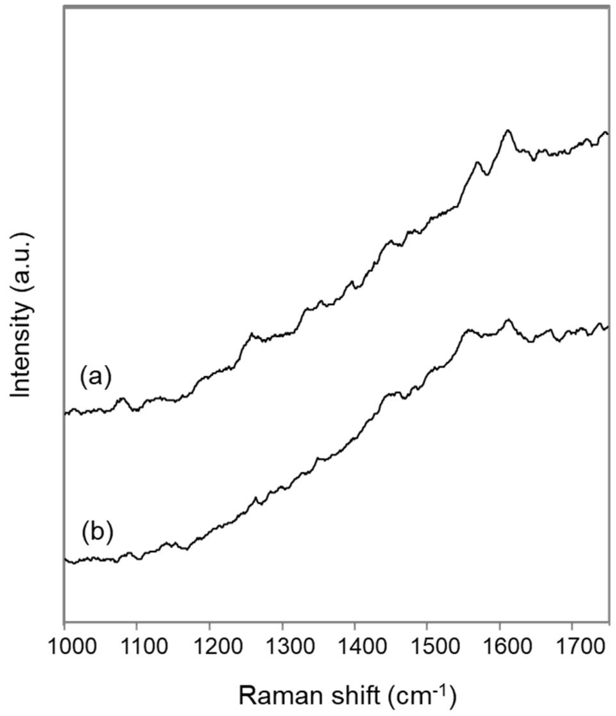

Figure S3. Micro-Raman spectra of the aqueous extracts obtained from the flowers of (a) *Commelina communis* and (b) *Salvia patens*, after purification on Sephadex LH-20 cartridges.
